# Global review of consumer preferences and willingness to pay for edible insects and derived products

**DOI:** 10.1016/j.gfs.2025.100834

**Published:** 2025-03

**Authors:** Zewdu Abro, Kibrom T. Sibhatu, Gebeyehu Manie Fetene, Mohammed Hussen Alemu, Chrysantus M. Tanga, Subramanian Sevgan, Menale Kassie

**Affiliations:** a International Centre of Insect Physiology and Ecology (*icipe*), Nairobi, Kenya; b Department of Economics & Institute of Development and Policy Research (IDPR), Addis Ababa University, Addis Ababa, Ethiopia; c Department of Food and Resource Economics, University of Copenhagen, Denmark

**Keywords:** Alternative protein sources, Entomophagy, Insect-based feed, Insect-based food, Sustainability, Circular economy

## Abstract

The rising global demand for protein, driven by population growth, urbanization, economic development, and climate change, underscores the need for sustainable alternative protein sources. Edible insects have emerged as a viable solution to enhance foodfeed and nutritional security while contributing to waste management. This study reviews 128 peer-reviewed publications to assess consumer preferences and willingness to pay (WTP) for edible insects and their derivatives. Findings reveal that studies predominantly focus on Europe and insect-based foods, with limited research on insect-based feeds and derived products. Consumers in Africa, Asia, and Latin America show higher acceptance of insect-based food compared to those in Western nations, where psychological barriers such as neophobia, disgust, and limited awareness hinder adoption. WTP for insect-based foods varies, with some consumers willing to pay a premium while others expect lower prices than conventional options. Integrating edible insects into sustainable food and feed ssytems requires public education, innovative marketing, and scaling up production.

## Highlights

• Global protein demand drives focus on edible insects for sustainable solutions.
• 79% of reviewed studies focus on Europe, with insect-based foods dominating research.
• WTP for insect-based food ranges from premiums to discount expectations.
• Acceptance of insect-based foods is higher in Africa, Asia, and Latin America.
• Awareness, neophobia, disgust, and knowledge gaps affect edible insect acceptance.

## 1 Introduction

The global demand for protein is rising rapidly, driven by population growth, urbanization, and economic development, particularly in low- and middle-income countries (OECD-FAO, 2023; da Costa et al., 2022; Drewnowski, 2024; Smith et al., 2024). Over the last decade, global per capita meat consumption has increased by 20%, with projections indicating this trend will continue (Revell, 2015; Stoll-Kleemann and O’Riordan, 2015). However, traditional protein sources face mounting challenges, including resource scarcity and depletion, public health implications, climate change, and their significant contribution to greenhouse gas emissions and deforestation (Fiala, 2008; Godfray et al., 2018; Poore and Nemecek, 2018; Revell, 2015; Steinfeld et al., 2006; Stoll-Kleemann and O’Riordan, 2015; FAO, 2019; Imathiu, 2020). These challenges underscore the need for an urgent transition to alternative, sustainable protein sources that can reshape food and feed systems while delivering environmental, health, and economic benefits.

Edible insects, traditionally consumed in Africa, Asia, and Latin America, are now gaining global recognition as a sustainable solution to meet the growing protein demand. They offer a viable pathway to alleviate protein and other nutrient deficiencies in human and animal diets while supporting the transition toward more sustainabile and resilience food and feed systems (Lange and Nakamura, 2023; Barragán-Fonseca, 2024; Bedsaul-Fryer et al., 2024; Colamatteo et al., 2024). In regions facing food insecurity, edible insects have the potential to diversify diets and combat hidden hunger (Imathiu, 2020; Adegboye et al., 2021; Babarinde et al., 2021; Van Huis et al., 2021; Lange and Nakamura, 2023). With over 2,200 insect species identified globally as suitable for consumption (Omuse et al., 2024), insects demonstrate significant advantages, including high feed conversion efficiency, short developmental lifecycle, and greater edible yields compared to conventional plant and livestock-based food sources (Klunder et al., 2012; Nowak et al., 2016; van Huis and Oonincx, 2017; van Huis and Oonincx, 2017; Higa et al., 2021; Omuse et al., 2024).

Insects are also nutritionally rich, providing protein, energy, healthy fats, fibers, and essential micronutrients (Van Huis, 2013; Nowak et al., 2016; Rumpold and Schlüter, 2013; Köhler et al., 2019; Imathiu, 2020; Van Huis et al., 2021). They have been used to address micronutrient deficiencies (Imathiu, 2020; Van Huis et al., 2021) and have been demonstrated to emit fewer greenhouse gases than traditional livestock (Oonincx et al., 2010; Kim et al., 2020; Pang et al., 2020; Parodi et al., 2020). Their farming demands less land, water, energy, and feed (Alexander et al., 2017; Kim et al., 2020; Pang et al., 2020; Parodi et al., 2020; Siddiqui et al., 2022; van Huis and Oonincx, 2017). Moreover, insects can efficiently convert waste into valuable biomass, contributing to a circular economy and environmental sustainability (van Huis and Oonincx, 2017; Nowak et al., 2016; Cappellozza et al., 2019; Ojha et al., 2020). This process provides multiple benefits, including food security, alternative animal feed, and nutrient-rich organic fertilizers that enhance crop growth, protection, and productivity (Beesigamukama et al., 2022; Ojha et al., 2020). Additionally, the insect sector creates green jobs, particularly for women and youth, thereby contributing to local economic development (Abro et al., 2020, 2022; Tanga et al., 2021; Verner et al., 2021).

The edible insect sector is expanding rapidly due to its nutritional and environmental benefits, growing consumer interest in sustainable protein options, and rising food and feed costs (Onsongo et al., 2018; Oonincx et al., 2020; Rumpold and Schlüter, 2013; Van Huis et al., 2021). Market forecasts project its value to reach $17.9 billion and 4.7 million tons by 2033, with a compound annual growth rate of 28.6% in value and 36.3% in volume from 2024 to 2033 (Meticulous Research, 2024).

Despite the environmental and societal benefits, consumer acceptance of edible insects, particularly insect-based foods, remains relatively low (Verneau et al., 2016; Rumpold and Langen, 2019; Onwezen et al., 2021; Bukchin-Peles, 2024; Meng et al., 2024). Understanding consumer preferences and willingness to pay (WTP) is essential for identifying marketing opportunities, enhancing product development that aligns with consumer needs, and bridging knowledge gaps. While prior studies have examined consumer and producer attitudes towards insects as food (e.g., Alemu and Olsen, 2020; Lombardi et al., 2019; Mustapa and Kallas, 2023; Sogari et al., 2022a, Sogari et al., 2022b; Spartano and Grasso, 2021a, 2021b; Verbeke, 2015; Cheseto et al., 2020) and feed (e.g., Chia et al., 2020; Domingues et al., 2020; Okello et al., 2023), the literature on consumer preference and WTP remains fragmented.

This paper synthesizes global evidence on consumer preferences and WTP for edible insects and insect-based products. It aims to support innovative edible insect business models, promote consumer acceptance, and stimulate further research and discussion on pathways to advance edible insect farming and its derived products within a circular economy framework.

Previous reviews have explored drivers and barriers to acceptance (Dagevos, 2021; Wassmann et al., 2021; Onwezen et al., 2021; Kröger et al., 2022; Talwar et al., 2024), strategies for promoting insect consumption (van Huis and Rumpold, 2023), and methodological approaches for assessing consumer acceptance (Sogari et al., 2019). Other reviews reported regional consumption patterns and utilization (Granados-Echegoyen et al., 2024), nutritional composition (Rumpold and Schlüter, 2013; Sánchez-Muros et al., 2014; Hlongwane et al., 2020), and species diversity (Caparros Megido et al., 2024; Omuse et al., 2024). However, these reviews lack a quantitative synthesis of empirical findings on consumer preferences and WTP. This review fills the gap by compiling detailed findings on the proportion of consumers willing to try (WTT) edible insects and their derived products, as well as their WTP.

## 2 Review methodology

### 2.1 Data sources and search strategy

**Fig. 1:**
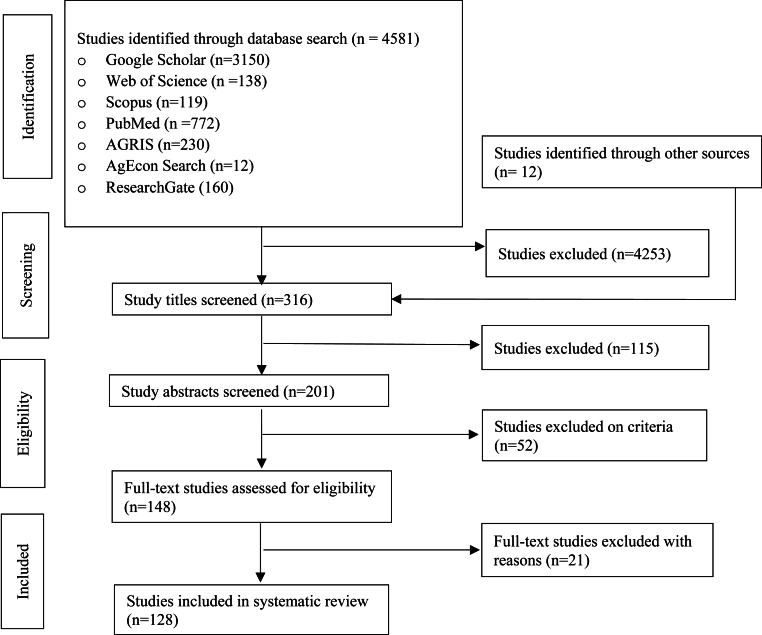
PRISMA flow diagram for selection and inclusion of studies.

This study uses a scoping review approach to synthesize articles published in peer-reviewed journals. This approach is widely accepted for identifying, mapping, and synthesizing key concepts, themes, and sources of evidence within a broad research topic (Munn et al., 2018). As shown in Fig. 1, the scoping review follows the Preferred Reporting Items for Systematic Reviews and Meta-Analyses (PRISMA) and Reporting Standards for Systematic Evidence Syntheses (ROSES) guidelines (Haddaway et al., 2018; Moher et al., 2009). We employ a structured search strategy that includes: (1) defining keywords and controlled vocabulary, namely, “edible insect(s),” OR “insect for food and feed,” OR “demand for insects,” OR “supply of insects,” OR “consumer attitude towards insects,” OR “willingness to eat insects,”OR “consumers’ willingness to eat insects,” OR “consumer willingness to pay for insect-based food,” OR “willingness to pay for insect food,” OR “willingness to pay for insect feed,”; (2) conducting searches in international research databases: Google Scholar, Web of Science, ResearchGate, Scopus, PubMed, and AgEcon Search; and (3) reviewing the titles, abstracts, and the keywords of articles. Moreover, references in the identified studies underwent thorough screening to identify any additional eligible studies.

### 2.2 Inclusion and exclusion criteria

The exclusion and inclusion criteria are summarized in Table 1. We include studies covering all periods and types of edible insects, those utilized for livestock and fish feed and human consumption, until January 31, 2024, without assessing the validity or quality of the studies. Our research yielded 4,581 results from the identified online databases. After rigorous screening of titles, abstracts, and full texts, we exclude studies that are solely literature reviews, non-peer-reviewed and unpublished reports, and those focusing on edible insects for pet feed (cat and dog). In the end, 128 studies retained for analysis.

**Table 1:** Inclusion and exclusion criteria.

	Inclusion	Exclusion
**Peer-review**	•Peer-reviewed journal articles and conferences	•Opinion articles, editorials, and NGO reports
**Focus areas**	•Edible insects for humans •Edible insects for livestock and aquatic feed	•Non-edible-related studies •Edible insects for pet feed
**Year**	•Published until January 2024	•After January 2024
**Keywords**	•“Edible insect(s),” OR “insect for food and feed,” OR “demand for insects,” OR “supply of insects,” OR “consumer attitude towards insects,” OR “willingness to eat insects,” OR “consumers' willingness to eat insects,” OR “consumer willingness to pay for insect-based food,” OR “willingness to pay for insect food” mentioned either in the title or abstract, keywords of the studies	•Not mentioned (either in the title, abstract, or keywords of the studies) of the words including “Edible insect(s),” OR “insect for food and feed,” OR “demand for insects,” OR “supply of insects,” OR “consumer attitude towards insects,” OR “willingness to eat insects,” OR “consumers' willingness to eat insects,” OR “consumer willingness to pay for insect-based food,” OR “willingness to pay for insect food”
**Methodology**	•Empirically grounded research •Reported original findings	•Not showing a clear research methodology •Opinionated arguments

### 2.3 Screening and data extraction

Using the Mendeley reference manager, we extracted, recorded, and removed duplicates (Elston, 2019). The inclusion of a paper was determined through assessments of its title, abstract, and full text. Subsequently, data extraction was carried out using an Excel spreadsheet. Given the breadth of the subject matter, we adopted a scoping review methodology to ensure inclusivity and comprehensive coverage. In addition, due to variations in the measurement of the outcome variables, our main analysis method is individual study reviews and narrations, which provide a better understanding of the findings of the reviewed studies.

### 2.4 Characteristics of reviewed studies

**Fig. 2:**
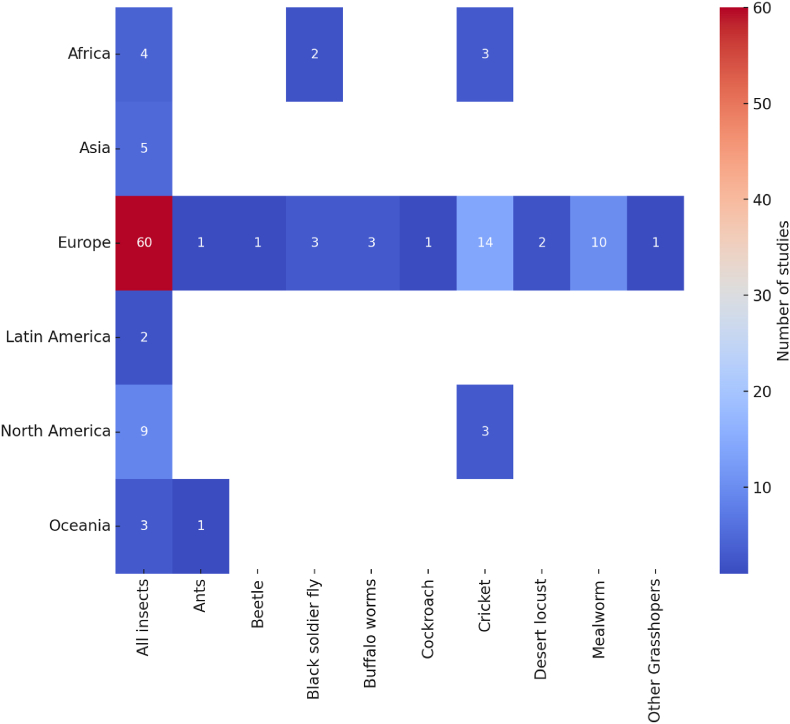
The number of reviewed studies (n = 128, data points = 383) focusing on insects for food, feed, or meat derived from animals fed insects-based diets.

Fig. 2 presents data from 128 studies examining consumer preferences and WTP for edible insects and insect-based products. Among these, 105 studies focus on the use of insects as food, 14 explore insect-based feed, and nine investigate preferences for animal products derived from livestock raised on insect-based feed. Together, these studies provide 383 data points. Sample sizes vary significantly, ranging from 32 to 1800 participants. To account for this variability, a weighted analysis approach based on sample size was employed. All reviewed studies were conducted within the past decade, with 94% published after 2015, indicating a growing interest in edible insect research.

The reviewed studies exhibit several notable characteristics, as detailed in Supplementary Material 1. The majority are concentrated in Europe and North America, likely reflecting high regional interest in edible insects driven by media, scientific inquiry, and policy discussions (Stull and Patz, 2020; Tanga and Kababu, 2023). This geographic concentration may also stem from limited research and investment capabilities in other regions. About 57% of studies assess consumer preferences and WTP for edible insects without specifying the exact insect type. Among studies that do specify, crickets (47%) and mealworms (25%) emerged as the most examined insects.

Regarding methodology, about 80% of the studies rely on hypothetical approaches, including contingent valuation, discrete choice experiments, and general questions about preferences and WTP for edible insects. The remaining 20% adopt non-hypothetical methodologies, employing sensory evaluations—where respondents taste edible insects—and actual purchase experiments, where participants pay for raw or processed insect products.

The studies reviewed demonstrate diverse approaches to presenting insect-based foods. About 47% focus on processed forms, such as insect powders, while 25% examine visible whole insects, often in combination with other foods. Around 12% of studies consider both whole and processed insects without distinguishing the results for each form. Additionally, 16% of the papers do not specify the form of consumption, whether processed or raw.

There is also variability in the measurement of outcome variables across the studies. Nearly half (49%) report the percentage of respondents willing to purchase insect-based products, while 42% use Likert scales to assess purchase willingness. A smaller proportion (9%) combine both methods. Most reviewed studies lack data on WTP, with only 7% of the 383 data points reporting the maximum amount respondents are willing to pay for edible insects.

## 3 Results and discussions

### 3.1 Preference for edible insects as food

**Fig. 3:**
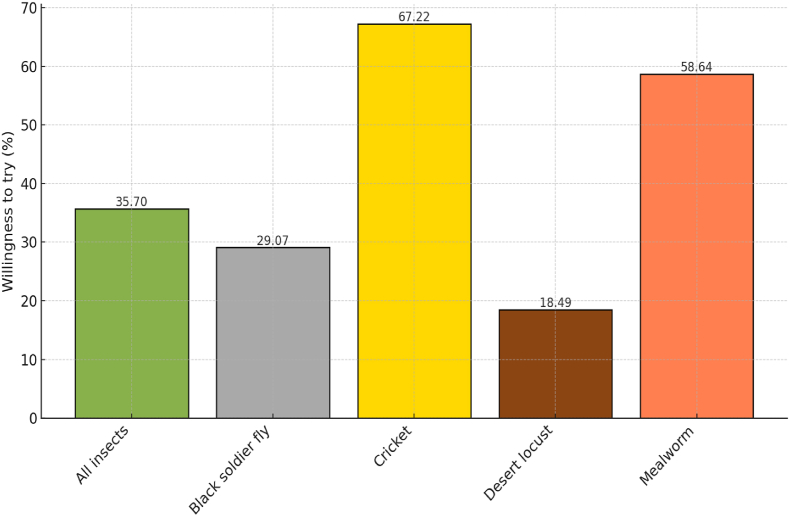
Willingness to try edible insects as food, measured as a percentage of participants. Data are based on 71 studies (151 data points).

The preference of respondents to try edible insects as food was analyzed across 71 studies, encompassing 151 data points (Fig. 3; supplementary material 2 provides details of each study). In cases where the type of insect was unspecified (categorized as “All insects" in Fig. 3), the proportion of respondents WTT insects was relatively low at 36%. Crickets (*Acheta domesticus*) and mealworms (*Tenebrio molitor*), however, were more widely accepted, reflecting global trends where crickets are widely promoted as the most popular insect-based food (Alemu and Olsen, 2020).

Fig. 3 also highlights a comparatively lower preference for black soldier fly (*Hermetia illucens*) (BSF) as food compared to cricket and mealworms. While BSF is primarily promoted as animal feed, Delicato et al. (2020) explored its potential as a human food ingredient. Their study involved students and staff at the University of Ghent in Belgium, who sampled baked goods, cakes, cookies, and waffles, where conventional butter was partially replaced with BSF fat at varying proportions (0%, 25%, and 50%). Participants were informed that BSF fat was included in the recipes but were not told the specific levels. Taste preferences were recorded following consumption, revealing that 22%–35% of respondents favored bakery products containing BSF fat, depending on the fat percentage.

Studies conducted in Europe and Africa assessed consumer preferences for desert locusts (*Schistocerca gregaria*) as an edible option (Fig. 3). Among the European studies, Berger and Wyss (2020) and De Boer et al. (2013) examined this preference alongside research in Africa by Cheseto et al. (2020). As shown in Fig. 3, about 18% of the respondents were willing to try desert locust-based food. This figure influenced by De Boer et al.’s study, where only 4% of the large sample of 1083 respondents favored locust-based snacks. Cheseto et al. reported that only 22% of 103 respondents in Kenya preferred locust-based oil cookies. In contrast, Berger and Wyss reported that about 46% of their respondents revealed a preference for whole desert locusts.

**Fig. 4:**
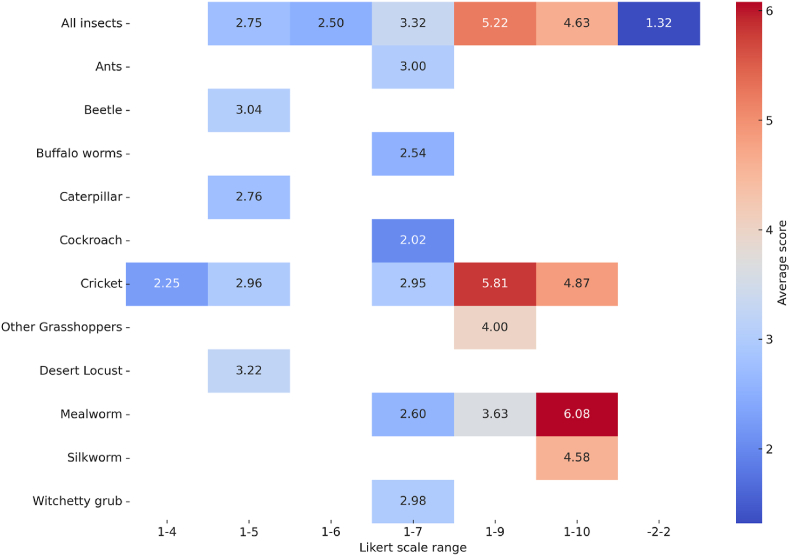
Willingness to try edible insects as food, measured using a Likert scale. Data are based on 40 studies (171 data points).

Fig. 4 provides insights into consumer preferences for edible insects assessed using the Likert scale. This analysis is based on data from 40 studies and 171 data points (see Supplementary Material 3). Because of variations in the Likert scales used across studies, willingness-to-try responses are reported separately. About 58% of the scores indicate neutral attitudes or hesitation about insect consumption, whereas 42% reflect positive perceptions, indicating a favorable outlook on insects as a food source.

Piha et al. (2018) utilized a 7-point Likert scale to evaluate consumer interest in insect-based foods in Finland, Sweden, Germany, and the Czech Republic. The scale ranged from 1 ('would definitely not buy') to 7 ('would definitely buy'). This study distinguished between whole insect meals and products containing processed edible insects, although the results for these categories were not reported separately. The average Likert scores, ranging between 2.7 and 3.3, indicate a relatively low willingness among consumers in these countries to purchase insect-based foods.

Some studies report a decrease in preference for insect-based foods as the insect content increases. For example, Alemu et al. (2017) conducted a study in rural and urban Kenya where buns were made by substituting wheat flour with 0%, 5%, and 10% cricket flour. Participants evaluated the taste of these buns using a 9-point Hedonic Likert scale. The average taste score revealed that buns with 5% cricket flour received the highest preference (6.8) compared to buns with no cricket flour (6.32) or with 10% cricket flour (6.33). Additionally, 80% of respondents liked the 5% cricket flour buns, whereas 74% and 67% expressed preference for buns with 0% and 10% cricket flour, respectively. Similarly, a study by Delicato et al. (2020) in Belgium observed a decrease in consumer acceptance as the proportion of BSF fat in food products increased. In the United States, Delgado et al. (2020) found that food containing 30% cricket powder was met with mixed reactions, with 15% of participants disliking its bitterness and hardness.

An ordinary least squares regression analysis was conducted to examine differences in WTT edible insects, considering factors such as demographics, insect consumption form (raw or processed), cultural background, and insect type (Table 2). The results indicate that respondents from Latin America were 19% more likely to express WTT than those in Europe. Further, when the WTT question specified a particular insect type, the average scores on 1–7 and 1–9 Likert scales were lower compared to the “All insects" category. Non-hypothetical studies reported higher preferences, although findings on the consumption form were inconclusive.

**Table 2:** Determinants of consumer willingness to try (WTT) edible insects for food.

	Linear regression of willingness to try insects for food measured in:				
		Likert scales			
Variables	Percent	1–10	1–5	1–7	1–9
Study participants were elite group (1/0) ^**a**^	5.359		−0.076	−1.260	2.260∗∗∗
	(7.194)		(0.161)	(1.038)	(0.000)
Non-hypothetical market (1/0)	8.545	1.837∗∗		2.057∗	2.305∗∗∗
	(13.639)	(0.564)		(1.107)	(0.000)
The insect in question was processed (1/0) ^**b**^	4.125		0.179∗	−1.376	1.225∗∗∗
	(4.969)		(0.073)	(1.026)	(0.000)
The insect in question was whole insect (1/0)	−1.871	−1.197∗∗∗	−0.428∗∗	−1.638	
	(5.481)	(0.317)	(0.146)	(0.993)	
Consumption form was not reported (1/0)	1.891	0.087		−1.395	
	(9.687)	(0.461)		(0.924)	
Cricket (1/0) ^**c**^	−21.555	0.367	2.209∗∗∗	0.493	−0.075∗∗∗
	(14.183)	(0.580)	(0.161)	(0.468)	(0.000)
Mealworm (1/0)	16.787		2.169∗∗∗	0.114	
	(10.264)		(0.161)	(0.592)	
Other insects (1/0)	−23.900		1.896∗∗∗	0.268	
	(17.737)		(0.161)	(0.428)	
Africa (1/0) ^**d**^	−3.900				2.450∗∗∗
	(9.067)				(0.000)
Asia (1/0)	3.673	1.346∗∗	1.637∗∗∗		
	(4.721)	(0.553)	(0.110)		
Latin America (1/0)	19.024∗∗∗				1.020∗∗∗
	(5.623)				(0.000)
North America (1/0)	14.834			−0.844	
	(9.948)			(0.941)	
Oceania (1/0)	3.340		0.568∗∗	0.391	
	(12.178)		(0.177)	(0.268)	
Constant	32.598∗∗∗	4.666∗∗∗	0.483∗∗∗	3.916∗∗∗	0.695∗∗∗
	(3.409)	(0.461)	(0.073)	(1.025)	(0.000)
Number of data points	151	38	22	83	18
Number of studies	71	8	6	17	6
R^2^	0.191	0.391	0.420	0.275	0.928

Note: ^**a**^the elite groups include respondents who were conference participants, professional athletes, students, and university staff. ^**b, c, d**^ the base groups, respectively, are studies where consumption (row or processed) was not asked (1/0), studies focusing on ‘all insects’ (1/0), and studies conducted in Europe (1/0). Clustered standard errors are shown in brackets. The number of data points corresponds to the frequency with which each study reported values for the outcome variables (WTT, measured in percentages and Likert scales).

### 3.2 Willingness to pay for insect-based foods

This section synthesizes findings from 11 studies that present consumers' maximum WTP for insect-based foods (refer to Supplementary Material 4). While most of these studies were conducted in Europe, two focused on African contexts. A significant fraction (45%) of the studies engaged participants in taste-testing of products made from crickets, mealworms, and BSF fat. Apart from Berger et al. (2018), insects presented in processed forms such as powders. The methods used to report WTP varied; some studies report results as percentage increases or reductions relative to conventional food prices, while others provided exact amounts the respondents willing to pay. In addition, summarizing WTP results is challenging due to differences in the food products tasted, such as cookies, meals, and buns.

In Africa, two studies conducted by Alemu and colleagues (Alemu et al., 2017; Alemu and Olsen, 2020) provided valuable insights. Alemu et al. report that respondents were willing to pay an additional 22–40 Kenyan Shillings (KES) for buns containing 5% cricket flour compared to buns without cricket flour. However, this premium decreased to 8–21 KES for buns with higher cricket flour content. Alemu and Olsen (2020) further examined how food tasting experience and peer influence affected WTP for buns with different cricket flour levels. Their study found that consumers were willing to pay an extra 22 KES for buns with 5% cricket flour compared to buns with 10%.

**Fig. 5:**
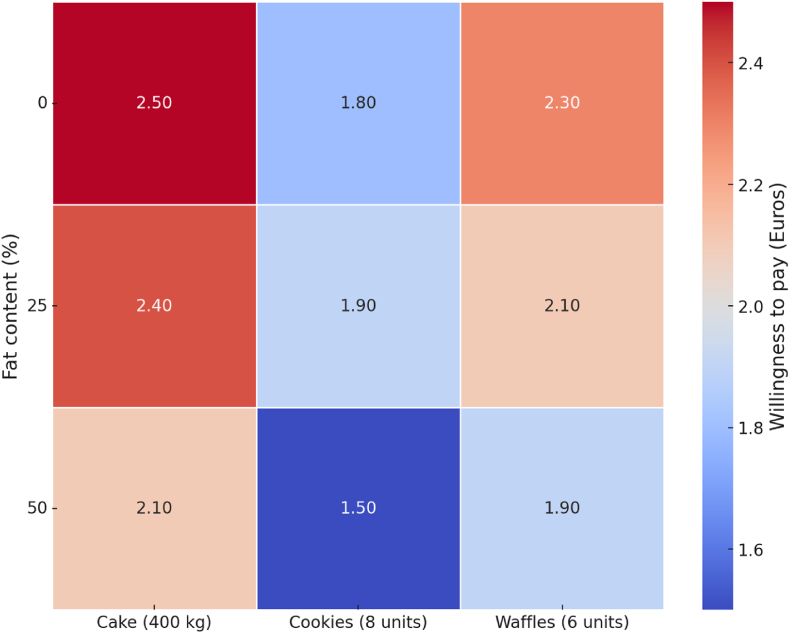
Willingness to pay for bakery products containing BSF fat. The authors developed this figure based on findings reported by Delicato et al. (2020).

The WTP for insect-based foods in Western countries showed mixed results. Kornher et al. (2019) reported that many participants were willing to pay prices comparable to conventional burgers. Conversely, Collins et al. (2019) reported that while some consumers were open to paying comparable prices to traditional protein foods, the majority were only willing to pay less than half. In Greece, Giotis and Drichoutis (2021) found that 43–76% of consumers required a 31–44% price reduction to consider buying energy bars and cookies containing insect-based flour. Delicato et al. (2020) further demonstrated that consumers' WTP decreases as the proportion of insect content in cakes and waffles increased (Fig. 5).

Research on WTP for insect-based foods has also examined how the provision of information affects consumer attitudes. For instance, Lombardi et al. (2019a) found that consumers tend to undervalue foods containing mealworms when no information about the benefits of edible insects is provided. However, with relevant information, the perceived value of such foods increases by 6–17% compared to foods without any benefit communication. Similarly, Michel and Begho (2023) reported a 36% rise in WTP when consumers were informed about the advantages of insect-based foods. de-Magistris et al. (2015) noted knowledge about the nutritional benefits of insect-based sushi led to an increase in WTP by €2.90, but the visible presence of insects in the food resulted in a significant reduction of WTP by €11.79. These findings underscore the importance of effective communication and presentation in enhancing consumer acceptance of insect-based foods.

### 3.3 Consumer preference and willingness to pay for animal products fed with insect-based feed

**Fig. 6:**
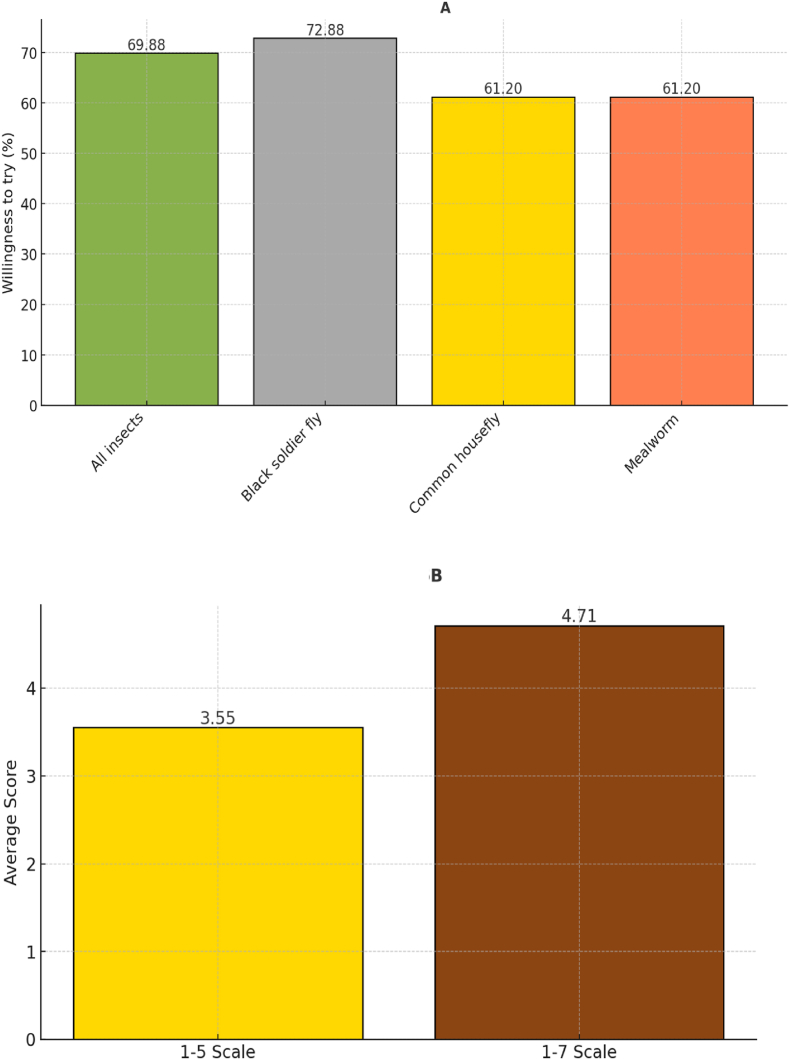
Willingness to consume animal products derived from insect-based feed. Fig. 6A represents data from 13 studies (26 data points). Fig. 6B represents data from 4 studies (12 data points).

Consumer preferences for meat and eggs from livestock fed on diets containing insect-based feed have been analyzed in 17 studies conducted in Europe (Supplementary Material 5). Among these, 13 studies assessed preferences by measuring the percentage of respondents WTT such products (Fig. 6A), while four used a Likert scale to evaluate preferences (Fig. 6B). Fig. 6A reveals that 61–73% of respondents expressed a willingness to try animal products from livestock raised on insect-based feed. Results from the Likert scale studies (Fig. 6B) align with these results, showing above-average consumer preferences for such products.

Among these studies, Spartano and Grasso (2021b) reported that 77% of UK consumers were willing to try eggs produced from livestock fed with insect-based feed. Barragán-Fonseca (2024) found that consumers considered fish fed with edible insects to be palatable, although their preference for such fish was lower compared to fish fed on conventional diets. Similarly, in France, 49–58% of respondents were willing to consume fish produced on insect-based feed (Bazoche and Poret, 2020).

**Fig. 7:**
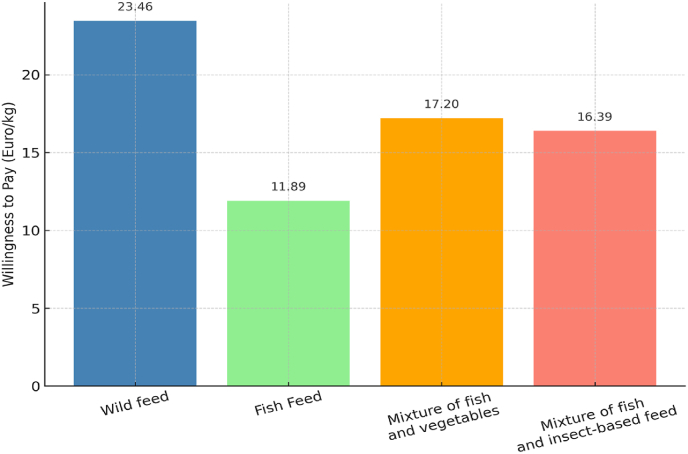
WTP for fillets derived from fish fed a diet containing edible insects. The authors developed this figure based on the findings reported by Llagostera et al. (2019).

When it comes to WTP for products from livestock fed on insect-based diets, Spartano and Grasso (2021b) revealed that UK consumers were willing to pay 18% more for such eggs compared to conventional ones. Llagostera et al. (2019) found a high WTP for fish fed on a combination of insect and fish meal (Fig. 7). However, Giotis and Drichoutis (2021) presented mixed findings in Greece, where 44% of consumers required a discount, while 55% were willing to pay a premium for fish raised on insect-based feed.

### 3.4 Preference and willingness to pay for insects-based feed

**Fig. 8:**
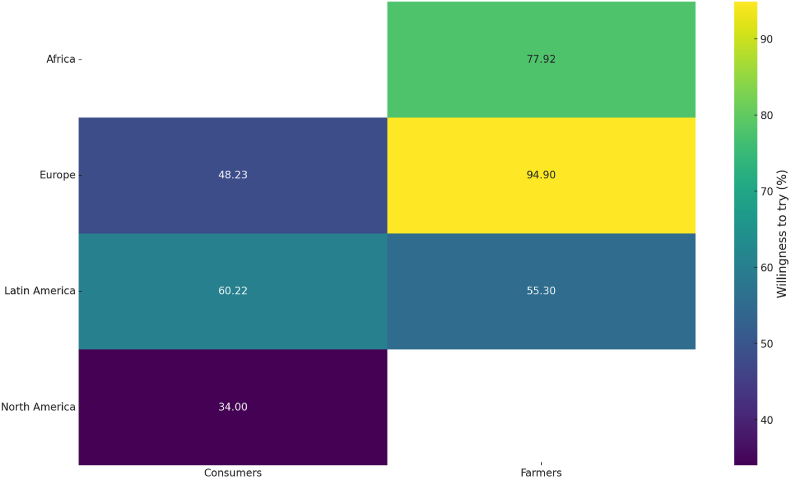
Willingness to try edible insects as feed, measured as a percentage of participants. Data are based on 13 studies (16 data points).

An analysis of 13 studies revealed a generally favorable attitude toward using insects as feed ingredients, with most respondents —predominantly farmers— expressing willingness to explore this option (see Supplementary Material 6 for details of the studies). A summary of the willingness to use edible insects for feed is presented in Fig. 8. Baldi et al. (2021) indirectly assessed preferences for insect-based feeds using a Likert scale. Respondents rated their agreement with statements such as “I find it natural for fish to feed on insects” and “I find it normal for farmed fish to be fed on insect-based feed” on a scale of 1–5. These statements received average scores of 3.87 and 3.57, respectively, indicating general acceptance of insect-based feeds among the participants.

In Kenya, two studies evaluated the WTP for insect-based feed among fish, pig, and poultry farmers (Chia et al., 2020; Okello et al., 2023). Chia et al. (2020) reported that 25–38% of the interviewed farmers had already incorporated insects into their animal feed. About 90% of farmers who valued insects as a feed ingredient were willing to pay a premium: 12% more for fish feed, 30% more for pig feed, and 16% more for poultry feed, compared to conventional feed options. These results were supported by Okello et al. (2023), whose choice experiment confirmed farmers were willing to pay higher prices for insect-based feed alternatives in Kenya.

## 4 Conclusions

### 4.1 The benefits of insects as food sources

This review highlights distinct regional differences in consumer attitudes toward insect-based foods. Consumers in Africa, Asia, and Latin America generally show greater openness to consuming insects compared to their Western counterparts, where reluctance (often associated with neophobia) remains prevalent. The WTP for insect-based foods also varies widely; some consumers are prepared to pay a premium, whereas others expect prices to be lower than conventional foods. Factors such as cultural practices, flavor, texture, awareness, and socio-demographics play a pivotal role in shaping consumer acceptance of edible insects (Hurd et al., 2019; Babarinde et al., 2021; Onwezen et al., 2021; Guiné et al., 2024; Sadowski et al., 2024; Bedsaul-Fryer et al., 2024; Omuse et al., 2024; Roccatello et al., 2024; Talwar et al., 2024). In many African, Asian, and Latin American cultures, insect consumption is deeply rooted in tradition (Hurd et al., 2019; Mulungu et al., 2023; Omuse et al., 2024). Beyond serving as dietary staples, insects in these societies are also valued for their medicinal and ceremonial roles. This cultural acceptance has enabled their seamless integration into everyday diets, a sharp contrast to Western societies, where insect consumption is less common (Verneau et al., 2016; Guiné et al., 2024; Hunter, 2024), leading to lower consumer awareness and acceptance. In Europe, limited awareness exists about edible insects’ high protein content and other nutritional advantages (Guiné et al., 2024). In contrast, in African, Asian, and Latin American regions, where insects have been part of traditional diets for centuries, awareness of their nutritional value is higher (Hurd et al., 2019; Krongdang et al., 2023; Mulungu et al., 2023; Omuse et al., 2024).

We identify several key entry points to promote the consumption and investment in edible insects. First, direct exposure through tasting experiences, which have been shown to impact consumer behavior positively (Alemu and Olsen, 2020; Collins et al., 2019; Sogari et al., 2018; Spartano and Grasso, 2021b). However, preference decreases with higher insect content in food products (Alemu et al., 2017; Delicato et al., 2020; de-Magistris et al., 2015). A gradual approach to incorporating edible insects into food products may help mitigate consumer resistance.

Second, overcoming psychological barriers such as neophobia and disgust, and knowledge gaps is vital for increasing acceptance (Collins et al., 2019; de-Magistris et al., 2015). Novel processing methods that reduce the visibility of insects in food, along with increasing awareness and governmental support, can boost demand (Giotis and Drichoutis, 2021; Higa et al., 2021). Aligning product development with cultural norms and emphasizing features like nutritional value and packaging are also essential strategies for promoting insects as a sustainable and alternative protein source (Hartmann and Siegrist, 2017; Mulungu et al., 2023; Bruckdorfer and Büttner, 2022; Pilianto et al., 2022). Anthropomorphic packaging, which incorporates human-like characteristics, emotions, or expressions into product designs, has proven effective in reducing neophobia and disgust and increasing consumer acceptance (Wang and Park, 2023; Meng et al., 2024).

Third, other strategies involve employing message framing to influence behavior and sharing information about edible insects' nutritional and environmental benefits (Verneau et al., 2016; Lombardi et al., 2019; Rumpold and Langen, 2019; Sogari et al., 2022a, Sogari et al., 2022b; Meng et al., 2024; Sadowski et al., 2024) and leveraging celebrity endorsements to promote insect-based products (Legendre and Baker, 2021; Park et al., 2022). Furthermore, targeting consumers motivated by novelty, sensory appeal, health, tasting experience, and environmental sustainability has been identified as an effective approach (Hwang et al., 2023; Kornher et al., 2019; Merlino et al., 2024; Begho, 2023; Mulungu et al., 2023).

Last, addressing price barriers remains critical, as edible insects are often priced higher due to limited production and niche market positioning (Ayieko et al., 2016; Han et al., 2017; House, 2016; Kornher et al., 2019; Niyonsaba et al., 2021; Talwar et al., 2024). Scaling up production, improving awareness, and increasing investments to advance the alternative protein market across different regions is essential to improve affordability (Talwar et al., 2024).

### 4.2 Insects as novel animal feed ingredient

The review demonstrates that consumers are more receptive to using edible insects as animal feed than as a human food source. This may be due to the perception that using insects in feed is more natural and aligns with existing practices (Sogari et al., 2022a, Sogari et al., 2022b). The growing market for insect-fed livestock products reflects this acceptance, although many consumers and producers remain unaware of the potential benefits of insects as feed. Those aware of their nutritional benefits are willing to pay a premium for such feeds. Despite the increasing demand for insect-based products and livestock raised on insect feed, a significant challenge lies in the capacity of the insect sector to meet this demand. While this review does not focus on supply-side issues, it is evident that the global insect farming industry must scale up production to support the rising interest in insect-based feed (Tanga and Kababu, 2023).

### 4.3 Limitations of current research and future directions

This section highlights key limitations identified during the review process. Many studies rely on hypothetical scenarios rather than observing actual consumer behaviors, which often result in discrepancies between stated intentions and real-world actions. Additionally, most research is concentrated in Europe and North America, with limited representation from other regions. Sample sizes are frequently small and biased toward specific groups, such as university affiliates or consumers already inclined toward novel foods, making it difficult to generalize findings to broader populations.

Research on insect-based food products is more extensive than research focusing on farmers' willingness to adopt and invest in insect-based feeds and animal products derived from such feeds. This is a critical gap, as scaling up production and integrating insects into feed and food systems requires understanding farmers’ and consumers' perspectives. Furthermore, the focus tends to be restricted to a limited number of insect species, neglecting the vast diversity of over 2200 species identified as having potential for human and animal consumption (Omuse et al., 2024). This lack of diversity may increasebiological and environmental risks, potentially disrupting food and feed production.

To address these gaps, future studies should move beyond hypothetical models and incorporate real-world consumer behavior through market-based experiments, retail analyses, and experimental auctions to generate actionable insights. Broader and more diverse samples across varied regional contexts are essential to improve representation. Expanding research to explore farmers' and consumers’ attitudes and barriers to using insect-based feeds, animal products derived from insect-based feed, and other insect-derived innovations is crucial for integrating these solutions into food and feed systems. Additionally, investigating a wider range of insect species and assessing their suitability for production, cultural acceptance, and market viability is critical for mitigating risks and maximizing opportunities.

Research into innovative marketing strategies and public education campaigns is also necessary to overcome psychological barriers, enhance acceptance, and improve understanding of edible insects' nutritional, environmental, and economic benefits. Aligning insect-based products with cultural practices and traditions can further increase acceptance. Scaling production and implementing supportive policies to improve affordability are essential steps toward mainstreaming edible insects. Furthermore, strengthening existing research is necessary to quantify the environmental benefits of insect farming, particularly its role in advancing circular economies and improving waste management systems.

## CRediT authorship contribution statement

**Zewdu Abro:** Writing – original draft, Methodology, Formal analysis, Data curation, Conceptualization. **Kibrom T. Sibhatu:** Writing – review & editing, Writing – original draft, Visualization, Validation, Methodology, Conceptualization. **Gebeyehu Manie Fetene:** Writing – original draft, Visualization, Methodology, Data curation, Conceptualization. **Mohammed Hussen Alemu:** Writing – original draft, Visualization, Validation, Methodology, Investigation, Data curation. **Chrysantus M. Tanga:** Writing – review & editing, Validation, Funding acquisition. **Subramanian Sevgan:** Writing – review & editing, Visualization, Validation, Resources. **Menale Kassie:** Writing – review & editing, Validation, Supervision, Resources, Funding acquisition.

## Declaration of competing interest

We declare there are no conflicts of interest.

## Data Availability

Data will be made available on request.
